# Predicting the Function and Subcellular Location of C*aenorhabditis Elegans* Proteins Similar to *Saccharomyces Cerevisiae * β-Oxidation Enzymes

**DOI:** 10.1002/1097-0061(20000930)17:3<188::AID-YEA27>3.0.CO;2-E

**Published:** 2000

**Authors:** Aner Gurvitz, Sigrid Langer, Martin Piskacek, Barbara Hamilton, Helmut Ruis, Andreas Hartig

**Affiliations:** 1 Institut für Biochemie und Molekulare Zellbiologie der Universität Wien and Ludwig Boltzmann Forschungsstelle für Biochemie Vienna Biocenter Dr Bohrgasse 9 Vienna A-1030 Austria; 2 Institute of Biochemistry and Molecular Cell Biology University of Vienna Vienna Biocenter, Dr Bohrgasse 9 Vienna A-1030 Austria

## Abstract

The role of peroxisomal processes in the maintenance of neurons has not been thoroughly investigated. We propose using *Caenorhabditis elegans* as a model organism for studying the molecular basis underlying neurodegeneration in certain human peroxisomal disorders, e.g. Zellweger syndrome, since the nematode neural network is well characterized and relatively simple in function. Here we have identified *C. elegans* PEX-5 (C34C6.6) representing the receptor for peroxisomal targeting signal type 1 (PTS1), defective in patients with such disorders. PEX-5 interacted strongly in a two-hybrid assay with Gal4p–SKL, and a screen using PEX-5 identified interaction partners that were predominantly terminated with PTS1 or its variants. A list of *C. elegans* proteins with similarities to well-characterized yeast β-oxidation enzymes was compiled by homology probing. The possible subcellular localization of these orthologues was predicted using an algorithm based on trafficking signals. Examining the C termini of selected nematode proteins for PTS1 function substantiated predictions made regarding the proteins' peroxisomal location. It is concluded that the eukaryotic PEX5-dependent route for importing PTS1-containing proteins into peroxisomes is conserved in nematodes. *C. elegans* might emerge as an attractive model system for studying the importance of peroxisomes and affiliated processes in neurodegeneration, and also for studying a β-oxidation process that is potentially compartmentalized in both mitochondria and peroxisomes.


**Yeast**
*Yeast* 2000; **17**: 188–200.

**Research Article**

### Predicting the function and subcellular location of *Caenorhabditis elegans* proteins similar to *Saccharomyces cerevisiae* β-oxidation enzymes

Aner Gurvitz *, Sigrid Langer, Martin Piskacek, Barbara Hamilton, Helmut Ruis and Andreas Hartig
*Institut für Biochemie und Molekulare Zellbiologie der Universität Wien and Ludwig Boltzmann Forschungsstelle für Biochemie, Vienna Biocenter, Dr Bohrgasse 9, A-1030 Vienna, Austria*

*Correspondence to: A. Gurvitz, Institute of Biochemistry and Molecular Cell Biology, University of Vienna, Vienna Biocenter, Dr Bohrgasse 9, A-1030 Vienna, Austria. http://www.at.embnet.org/bmz/ord1.htm
E-mail: AG@abc.univie.ac.at*

Received: 30 May 2000
Accepted: 14 July 2000

### Abstract

**The role of peroxisomal processes in the maintenance of neurons has not been thoroughly investigated. We propose using *Caenorhabditis elegans* as a model organism for studying the molecular basis underlying neurodegeneration in certain human peroxisomal disorders, e.g. Zellweger syndrome, since the nematode neural network is well characterized and relatively simple in function. Here we have identified *C. elegans* PEX-5 (C34C6.6) representing the receptor for peroxisomal targeting signal type 1 (PTS1), defective in patients with such disorders. PEX-5 interacted strongly in a two-hybrid assay with Gal4p–SKL, and a screen using PEX-5 identified interaction partners that were predominantly terminated with PTS1 or its variants. A list of *C. elegans* proteins with similarities to well-characterized yeast β-oxidation enzymes was compiled by homology probing. The possible subcellular localization of these orthologues was predicted using an algorithm based on trafficking signals. Examining the C termini of selected nematode proteins for PTS1 function substantiated predictions made regarding the proteins' peroxisomal location. It is concluded that the eukaryotic PEX5-dependent route for importing PTS1-containing proteins into peroxisomes is conserved in nematodes. *C. elegans* might emerge as an attractive model system for studying the importance of peroxisomes and affiliated processes in neurodegeneration, and also for studying a β-oxidation process that is potentially compartmentalized in both mitochondria and peroxisomes. Copyright © 2000 John Wiley & Sons, Ltd.**

**Keywords:** ***Caenorhabditis elegans*; *Saccharomyces cerevisiae*; β-oxidation; peroxisome; PTS1; PEX-5; tetratricopeptide repeat (TPR); two hybrid**

### Introduction

The recent completion of the nematode genome sequencing project (Consortium, 1998) promises to provide valuable insights into hitherto uncharacterized biochemical processes in *Caenorhabditis elegans* that have already been extensively studied in *Saccharomyces cerevisiae* (Chervitz *et al.*, 1998), such as β-oxidation of fatty acids. The β-oxidation process occurs in the peroxisomal compartment of all eukaryotic systems tested and, additionally, in the mitochondria of mammalian cells (Kunau *et al.*, 1995). Peroxisomes are organelles demarcated by a single membrane that characteristically contain enzymes for oxidative reactions involving molecular oxygen and for metabolizing hydrogen peroxide (de Duve and Baudhuin, 1966).

Peroxisomal matrix enzymes, including those involved in β-oxidation, are imported with the help of specific targeting signals that interact with cognate receptors. The type 1 peroxisomal targeting signal (PTS1) of some proteins is a carboxy-terminal tripeptide that consists of SKL or a variant thereof (Gould *et al.*, 1987; Subramani, 1993), i.e. S/A/C–K/R/H–L (McNew and Goodman, 1996). PTS1 is recognized by Pex5p (Van der Leij *et al.*,

Copyright © 2000 John Wiley & Sons, Ltd.

1993) in all organisms studied to date. Pex5p has been shown to accommodate considerable degeneracy at the PTS1 (Sommer *et al.*, 1992; Elgersma *et al.*, 1996; Kragler *et al.*, 1998), and to depend on additional residues adjacent to the C-terminal tripeptide for its binding affinity to cargo protein (Lametschwandtner *et al.*, 1998). A second peroxisomal protein targeting signal, PTS2, consists of the sequence R/K–L/I–$X_5$–H/Q–L (McNew and Goodman, 1996). The PTS2 receptor is represented by Pex7p (Marzioch *et al.*, 1994; Rehling *et al.*, 1996). PTS2 is cleaved off in some instances, e.g. rat thiolase and watermelon malate dehydrogenase, but not in others, e.g. yeast thiolase and amine oxidase (Subramani, 1993). Some peroxisomal matrix enzymes do not contain either of the two known targeting signals (Karpichev and Small, 2000).

Peroxisomal diseases in humans often lead to degeneration or poor development of neurons. For example, Zellweger syndrome representing a class of peroxisome biogenesis disorders in which the entire peroxisomal compartment is compromised is associated with severe neurological impairments; PEX5 is defective in a number of Zellweger patients (Online Mendelian Inheritance in Man, OMIM$^{TM}$, Johns Hopkins University, Baltimore, MD; http://www.ncbi.nlm.nih.gov/omim/, OMIM number 214100: 6/4/1998). Neurological abnormalities can also be caused by single peroxisomal *β*-oxidation enzyme deficiencies, such as peroxisomal 3-oxoacyl-CoA thiolase deficiency (OMIM number 261510: 4/25/2000), peroxisomal L-bifunctional protein (OMIM number 261515: 3/30/1999) and peroxisomal D-bifunctional protein (OMIM number 601860: 9/30/1999; Itoh *et al.*, 2000). Since the neural development of *C. elegans* has been characterized (Sulston *et al.*, 1983), the nematode might serve as a model organism for studying the possible roles of peroxisomes and the biochemical processes contained therein in the development and maintenance of neurons.

A further reason for considering nematodes as a better alternative to fungal models for investigating fatty acid degradation lies in the subcellular distribution of *β*-oxidation. In fungi, *β*-oxidation is strictly a peroxisomal process (Kunau *et al.*, 1995). Nematodes might turn out to resemble mammals by possessing both peroxisomal and mitochondrial *β*-oxidation, and could therefore serve as a more appropriate model system than *S. cerevisiae* for studying the relative contribution of the two compartments to fatty acid breakdown. *C. elegans* peroxisomes have been shown previously to contain catalase (Maebuchi *et al.*, 1999; Togo *et al.*, 2000), an enzyme intimately associated with $H_2O_2$-generating enzymes, among them acyl-CoA oxidase, involved in peroxisomal fatty acid *β*-oxidation. However, it is hitherto unclear whether: (a) the conjectured *C. elegans β*-oxidation process is compartmentalized in peroxisomes; and (b) as in mammals, the process hypothesized to occur in nematode peroxisomes is augmented by a mitochondrial one.

In this study we considered *C. elegans* as a model system for investigating the possible connection between peroxisomal processes and neuronal development. To assess whether nematode peroxisomes potentially accommodate *β*-oxidation enzymes, the *C. elegans* databases were searched for putative orthologues of the PTS1 receptor, and for proteins similar to *S. cerevisiae* enzymes with *β*-oxidation functions. By additionally searching for the presence of PTS1, PTS2, and mitochondrial leader sequences in these nematode proteins using the PSORT II algorithm, a list was compiled representing candidate peroxisomal and mitochondrial *β*-oxidation enzymes. To underscore the algorithm predictions as to which of the nematode proteins could represent a peroxisomal protein due to a potential PTS1, *C. elegans* PEX-5 was used to analyse C-terminal tripeptides occurring on selected nematode proteins in two-hybrid interaction assays.

### Materials and methods

### Strains, plasmids and transformations

*Escherichia coli* strain DH10B was used for all plasmid amplifications and isolations. The *S. cerevisiae* strains and plasmids used here are described in Table 1. The yeast two-hybrid strain PCY3 is a derivative of PCY2 (Chevray and Nathans, 1992). Transformations of yeast strains were performed according to Chen *et al.* (1992).

### Growth conditions

Yeast transformants were selected on minimal medium (SC) containing 0.67% (w/v) Yeast Nitrogen Base without amino acids, 2% (w/v) D-glucose, 2% (w/v) agar, and supplemented with bases and amino acids (20–150 μg/ml) as required

Copyright © 2000 John Wiley & Sons, Ltd.

(SC–Leu–Trp). For quantitative measurements of β-galactosidase activity, yeast cells were grown overnight at 30°C with shaking in liquid selective medium (as above but without agar), diluted to $A_{600}=0.2$ in the same medium, and collected after 6–8 h at $A_{600}=1$.

### Computer-based predictions

The amino acid sequences of *S. cerevisiae* peroxisomal proteins were obtained from YPD™ (Costanzo *et al.*, 2000), and compared using the BLAST programme (Altschul *et al.*, 1997) with the worm database WormPD™ (http://www.proteome.com/databases/index.html). Each nematode protein was then analysed using the PSORT II programme for predicting the subcellular sites of proteins based on their amino acid sequences (http://www2.imcb.osaka-u.ac.jp/psort/form2.html). The peroxisomal matrix-targeting sequences used by the PSORT II programme were based on the published consensus for PTS1 and PTS2 (McNew and Goodman, 1996), as noted in the Introduction. For predicting mitochondrial proteins, PSORT II employed a method that recognizes mitochondrial targeting signals based on the amino acid composition of the N-terminal 20 residues. The PSORT II users' manual does not specify the algorithm's reasoning system in a situation in which a protein could contain both a mitochondrial and a peroxisomal targeting signal. The values in the tables represent the percentage probability for any given protein to be located in a particular subcellular site.

### Molecular cloning of *C. elegans pex-5*

The oligonucleotides used here are listed in Table 1. General nucleic acid manipulations were done according to standard protocols (Sambrook *et al.*, 1989). Purification of DNA following agarose electrophoresis was performed using QIAEX II (Qiagen Inc., Valencia, CA). Nematode cDNA was made by applying reverse transcription to purified nematode RNA using the 3′-end oligonucleotide H514, according to the manufacturer's protocol. To obtain the cDNA for the complete nematode PEX-5 (502 amino acids), polymerase chain reaction (PCR) was applied to cDNA obtained from the previous reverse transcription reaction using 50 pmol each of oligonucleotides H513 and H514 at an annealing temperature of 50°C (25 cycles) and a 1 : 9 mixture of *Pfu* and *Taq* DNA polymerases. This resulted in a single 1.5 kb DNA fragment that was digested with *Kpn*I (cutting within the nucleotide sequence of H513) and *Sph*I (cutting within H514). The digested DNA was ligated into a similarly digested plasmid vector pUC18, resulting in plasmid pSL5. Automatic nucleotide sequencing verified that no mutations were introduced into the PCR product.

To obtain the C-terminal half (291 amino acids) of nematode PEX-5, a similar approach was followed using oligonucleotides H512 and H514. An *Eco*RI- and *Sph*I-doubly digested PCR product was ligated to a similarly cut pUC18, resulting in plasmid pSL2. Nucleotide sequencing revealed a TTA to TTG wobble mutation at the Leu codon representing amino acid position 306 in PEX-5. To generate a two-hybrid construct encoding the tetratricopeptide repeat domain of PEX-5 in pGBT9 (Bartel *et al.*, 1993), PCR was applied to pSL2 template DNA using oligonucleotides H542 and H543. The resulting 0.8 kb PCR product encoding 267 amino acids of the PEX-5 C terminus (amino acid positions 215–481) was digested with *Eco*RI and *Bam*HI and inserted into a similarly digested pGBT9, resulting in pSL8. The insert was verified by automatic sequencing.

### Two-hybrid screen and enzyme assays

Two-hybrid experiments were conducted as described (Lametschwandtner *et al.*, 1998) according to the Matchmaker two-hybrid system protocol (Clontech Laboratories, Inc., Palo Alto, CA). A pSL8-containing yeast strain PCY3 (ySL1) was additionally transformed using a pGAD GH-derived peptide library consisting of 16 NN(T/G) codons fused to the activation domain of yeast Gal4p (Yang *et al.*, 1995). Transformants were replica-plated to selective medium containing 5-bromo-4-chloro-3-indolyl-β-D-galactopyranoside (X-gal), and blue colonies were isolated for further analysis. Library plasmids from blue single colonies were isolated and verified by reintroduction to ySL1 cells. β-Galactosidase activity was confirmed on X-gal plates, and the corresponding library plasmids sequenced. For two-hybrid interactions, β-galactosidase activity measurements were conducted using *O*-nitrophenyl-β-D-galactoside, as described (Miller, 1972). Protein concentrations were determined according to the method of Bradford (1976). Values reported here represent the mean of three independent experiments per strain, ± standard deviation.

Copyright © 2000 John Wiley & Sons, Ltd.

Table 1. *S. cerevisiae* strains, plasmids and oligonucleotides used

| Strain, plasmid or oligonucleotide | Description | Source or reference |
|---|---|---|
| *Strains*[a] | | |
| (1) PCY3 | *MATα his3-Δ200 ade2-101[ochre] trp1-Δ63 leu2 gal4Δ gal80Δ LYS2::GAL1-HIS3 URA3::GAL1-lacZ* | (Chevray and Nathans, 1992) |
| (2) yGL1[1] | Containing plasmid pAH987 | (Lametschwandtner *et al.*, 1998) |
| (3) ySL1[1] | Containing plasmid pSL8 | This study |
| yAG1254[2] | Containing plasmid Sc01 | This study |
| yAG1255[2] | Containing plasmid PL1_4 | This study |
| yAG1256[3] | Containing plasmid Sc01 | This study |
| yAG1257[3] | Containing plasmid PL1_4 | This study |
| yAG1262[3] | Containing plasmid Sc07 | This study |
| yAG1263[3] | Containing plasmid Sc08 | This study |
| yAG1264[3] | Containing plasmid Sc09 | This study |
| yAG1265[3] | Containing plasmid Sc10 | This study |
| yAG1266[3] | Containing plasmid Sc18 | This study |
| yAG1267[3] | Containing plasmid Sc22 | This study |
| yAG1268[3] | Containing plasmid Sc24 | This study |
| yAG1269[3] | Containing plasmid Hs40 | This study |
| yAG1270[3] | Containing plasmid Sc26 | This study |
| yAG1271[3] | Containing plasmid Sc27 | This study |
| *Plasmids* | | |
| pSL5 | cDNA for the complete *C. elegans* PEX-5 (502 aa) in pUC18 | This study |
| pSL2 | cDNA for the C-terminus of *C. elegans* PEX-5 (291 aa) in pUC18 | This study |
| pSL8 | pGBT9 containing the cDNA for *C. elegans* PEX-5 TPR domain (267 aa) | This study |
| pAH987 | pGBT9 containing the DNA for *S. cerevisiae* Pex5p TPR domain (305 aa) | (Lametschwandtner *et al.*, 1998) |
| PL1_4 | pGAD GH expressing Gal4p-FEWGSQGWTRGRMHML | (Lametschwandtner *et al.*, 1998) |
| Sc01 | pGAD GH expressing Gal4p-CERSKL | (Lametschwandtner *et al.*, 1998) |
| Sc07 | pGAD GH expressing Gal4p-QRKANGRDRGGWWAKL | (Lametschwandtner *et al.*, 1998) |
| Sc08 | pGAD GH expressing Gal4p-RQRELSANALGGLAKL | (Lametschwandtner *et al.*, 1998) |
| Sc09 | pGAD GH expressing Gal4p-NGMTRSGRQGGGFAKL | (Lametschwandtner *et al.*, 1998) |
| Sc18 | pGAD GH expressing Gal4p-SGGVVARAAKM | (Lametschwandtner *et al.*, 1998) |
| Sc22 | pGAD GH expressing Gal4p-TWNRETKGLNAVYGKL | (Lametschwandtner *et al.*, 1998) |
| Sc24 | pGAD GH expressing Gal4p-GKNRGSESHGSARQKL | (Lametschwandtner *et al.*, 1998) |
| Sc26 | pGAD GH expressing Gal4p-KRVWRRQWSTGRKLKL | (Lametschwandtner *et al.*, 1998) |
| Sc27 | pGAD GH expressing Gal4p-WYGPGPGCCRRRDLKL | (Lametschwandtner *et al.*, 1998) |
| Hs40 | pGAD GH expressing Gal4p-EGLIVMLERGKL | (Lametschwandtner *et al.*, 1998) |
| pGBT9 | Two-hybrid construct containing the Gal4p DNA binding domain | (Bartel *et al.*, 1993) |
| pGAD424 | Corresponding two-hybrid construct with the Gal4p activation domain | (Bartel *et al.*, 1993) |
| *Oligonucleotides* | | |
| H512 | 5′-AGGAGAATTCGCAAACCCTTTCACAACCATGTCAG-3′ | This study |
| H513 | 5′-AGGAGAGGTACCATGAAAGGAGTTGTAGAAGGACAATG-3′ | This study |
| H514 | 5′-GAAGAGGCATGCGGATCCCTAGACTAGAGAGGCTTTTACAGC-3′ | This study |
| H542 | 5′-AGGAGAATTCGCAGGTAGCGGTAGCAATTTCACAACCATGTCAG ATCCAC-3′ | This study |
| H543 | 5′-GAAGAGGCATGCGGATCCCTAGACATTCGATGTTCGGATTGCTGC-3′ | This study |

[a]The numbers in superscript following the strains' designations refer to their parental genotypes, e.g. yGL1[1] was derived from (1) PCY3. aa = amino acids. TPR = tetratricopeptide repeat

### Results

### Identification of *C. elegans* PEX-5

Import of most yeast proteins into the peroxisomal matrix depends on Pex5p (Van der Leij *et al.*, 1993). One of the basic criteria for the predictions made in the course of this study using the PSORT II algorithm was the potential presence or absence of the cognate signal PTS1 in candidate β-oxidation enzymes. The consensus for PTS1 is no longer

Copyright © 2000 John Wiley & Sons, Ltd. 

precise since non-canonical C-terminal tripeptides can function as PTS1 due to degeneracy and to neighbouring amino acid residues acting as PTS1 enhancers (Elgersma *et al.*, 1996; Lametschwandtner *et al.*, 1998). Hence, relying alone on an algorithm recognizing the strict consensus might exclude potential peroxisomal matrix proteins terminating with non-canonical PTS1s.

To gain a better understanding of whether proteins predicted by computer algorithms to contain PTS1s potentially represented peroxisomal enzymes, the C termini of such candidates could be examined for PTS1 function. This could be done by assaying for interactions with the cognate Pex5p, provided of course that the mechanism of protein import is conserved in the organism under investigation. For example, previous yeast two-hybrid experiments using *S. cerevisiae* Pex5p predicted that a non-conserved C-terminal tripeptide HRL might function as PTS1 (Lametschwandtner *et al.*, 1998). This prediction was subsequently validated by the discovery of a yeast peroxisomal protein Eci1p terminating with this tripeptide (Gurvitz *et al.*, 1998; Geisbrecht *et al.*, 1998), albeit the protein is proposed to contain an additional cryptic PTS (Karpichev and Small, 2000).

A search of the *C. elegans* databases for proteins with similarities to yeast Pex5p revealed a putative protein, C34C6.6, that additionally showed a strong similarity to all other known PTS1 receptors, including the most recently identified *Leishmania donovani* counterpart (Jardim *et al.*, 2000). Since yeast Pex5p (612 amino acids) was more similar to the 502 amino acid-long protein C34C6.6 (28% identity, 44% similarity over a length of 471 amino acids; WormPD$^{TM}$) than to other nematode proteins with tetratricopeptide repeat motifs, this meant that C34C6.6 could represent nematode PEX-5.

To confirm the identity of C34C6.6 as PEX-5, the open reading frame for this novel protein was amplified by applying polymerase chain reaction to nematode cDNA. The nucleotide sequence of the resulting DNA fragment matched that of C34C6.6 cDNA. A fragment representing the C-terminal half of the putative PEX-5 (267 amino acids), which included these tetratricopeptide repeat motifs, was ligated to the appropriate vector for two-hybrid interaction studies, and two-hybrid assays were performed on Gal4p-activating domains extended at their respective carboxy-termini with either SKL or HML. These and subsequent Gal4p fusion proteins used differed from one another only with respect to their C-terminal 16 (or less) amino acids (Table 1). Although these fusions presumably make available for interaction with PEX-5 additional amino acids, current knowledge stipulates that PTS1 is confined to the C termini of peroxisomal proteins. Hence, in the present analyses only the terminal three to four amino acid residues were considered.

The rationale for choosing SKL as a positive interacting partner was fairly straightforward, since it represents the canonical PTS1 in practically all organisms tested (Gould *et al.*, 1987, 1990). The C-terminal tripeptide HML, representing a less efficient PTS1, was chosen as an arbitrary control since it does not meet the strict consensus and was therefore expected to interact significantly less efficiently with the Pex5p-like protein. As a comparison, these SKL- and HML C termini were also assayed using yeast Pex5p (Table 2). Measurements of β-galactosidase activities demonstrated that, like the yeast PTS1 receptor, C34C6.6 also interacted with SKL to an approximately four-fold greater extent than with HML (Table 2), thereby arguing in favour of its function as nematode PEX-5.

To emphasize the function of C34C6.6 as the nematode PTS1 receptor, a two-hybrid screen was conducted using a protein library consisting of Gal4p extended by random C-termini in a similar manner to that previously performed with the yeast and human counterparts (Lametschwandtner *et al.*,

Table 2. Comparison of two-hybrid interactions between *C. elegans* PEX-5 or *S. cerevisiae* Pex5p and SKL- or HML-extended protein partners

| Strain | C terminus | β-Galactosidase activity[a] | Relative level |
|---|---|---|---|
| *C. elegans* PEX-5 | | | |
| yAG1255 | M-HML[b] | 123.8 ± 24.0 | 1.0 |
| yAG1254 | R-SKL | 556.8 ± 5.2 | 4.5 |
| *S. cerevisiae* Pex5p | | | |
| yAG1257 | M-HML | 39.0 ± 1.6 | 1.0 |
| yAG1256 | R-SKL | 162.6 ± 9.7 | 4.2 |

[a]nmol *O*-nitrophenyl-β-D-galactoside hydrolysed/min/mg protein; mean ± SD, $n = 3$.
[b]The amino acid to the left of the hyphen is adjacent to the C-terminal tripeptide and may be important for PTS1 function (Lametschwandtner *et al.*, 1998).

Copyright © 2000 John Wiley & Sons, Ltd.

Table 3. C termini of Gal4p proteins interacting with *C. elegans* PEX-5

| Canonical PTS1s[a] | | Non-canonical PTS1s[b] | | | | Other C-terminal tripeptides | |
|---|---|---|---|---|---|---|---|
| SKL | 5 | SSL | 2 | GKL | 1 | ANV | 1 |
| AKL | 8 | HKL | 2 | AKV | 1 | ASM | 1 |
| ARL | 7 | CSL | 2 | AAL | 1 | FKM | 1 |
| SRL | 1 | SML | 2 | AML | 1 | GNL | 1 |
| CKL | 1 | ASL | 2 | SKM | 1 | | |
| Total | 22 | | | | 15 | | 4 |

[a]PTS1s conforming to the consensus S/A/C-K/R/H-L (McNew and Goodman, 1996).
[b]PTS1s not fully conforming to this consensus, but to the 'two out of three' rule (Elgersma *et al.*, 1996).

1998). Verified library plasmids obtained from a representative batch of blue single colonies were sequenced and the corresponding C-termini deduced. The results (Table 3) demonstrated that C34C6.6 interacted preferentially with canonical PTS1s (22) as well as non-canonical ones (15) compared with other C termini (4), bearing favourably on our conjecture that it represents nematode PEX-5. These results were generally very similar to those previously obtained using screens based on the human, yeast or plant PTS1 receptors (Kragler *et al.*, 1998; Lametschwandtner *et al.*, 1998), in that most of the interacting C termini turned out to be either canonical or non-canonical PTS1s. An exhaustive two-hybrid screen using *C. elegans* PEX-5 is the subject of current investigation, and is therefore not discussed further. With this PEX-5 two-hybrid construct at hand it was possible to verify experimentally the algorithm predictions made in the following sections regarding PTS1-terminating nematode proteins that could be involved in *β*-oxidation.

### *C. elegans* orthologues of classical *β*-oxidation enzymes: Pox1p-like proteins

The four *S. cerevisiae* enzyme activities of 'classical' *β*-oxidation include Pox1p, acyl-CoA oxidase; Fox2p, 2-enoyl-CoA hydratase 2 and D-specific 3-hydroxyacyl-CoA dehydrogenase; and Pot1p/Fox3p, 3-ketoacyl-CoA thiolase (Dmochowska *et al.*, 1990; Hiltunen *et al.*, 1992; Igual *et al.*, 1991; Einerhand *et al.*, 1991). To identify potential Pox1p orthologues, a search of the *C. elegans* database was performed. Although *S. cerevisiae* contains only one acyl-CoA oxidase (whose import route has not yet been identified), other yeast species contain several peroxisomal isoenzymes. For example, the yeast *Yarrowia lipolytica* contains at least five *POX* genes (Wang *et al.*, 1999).

The result of the computer-based search for Pox1p-like nematode proteins is shown in Table 4. As expected, the PSORT II algorithm predicted those worm proteins terminating with SKL to be primarily peroxisomal (Table 4). Of the remaining proteins, the AKM-ending Pox1p orthologue was predicted to be a cytosolic protein (Table 4), whereas those orthologues terminating with AKL, a canonical PTS1, were predicted to be peroxisomal or mitochondrial (although they all contain identical 15-mer carboxyl termini; WormPD®). To test the validity of the computer-based predictions regarding the PTS1 function of these tripeptides, nematode PEX-5 was used to examine two-hybrid interactions with representative C termini extending from the Gal4p activation domain (Table 5).

The two-hybrid results demonstrated that AKL-terminating Gal4p proteins interacted with the nematode PEX-5 1.6–6.6-fold more efficiently than the protein ending with HML, whereas the AKM terminus tested was 1.7-fold more efficient than HML (upper two clusters, Table 5). This variation in interaction efficiencies among the AKL termini could have been due to specific amino acids at other positions in the proteins tested (Kragler *et al.*, 1998; Lametschwandtner *et al.*, 1998). The relatively high probabilities for the AKL-terminating proteins F08A8.2 and F08A8.3 to be mitochondrially located was due to amino acid sequences in their respective N termini that potentially represented mitochondrial leader sequences. Although not specified in the users' manual, it appears that in cases of dual targeting, the PSORT II algorithm allocates the protein preferentially to the mitochondria. In summary, the BLAST and PSORT II data in Table 4 combined with the two-hybrid results in Tables 3 and 5 were congruent with our hypothesis that nematodes might contain a peroxisomal acyl-CoA oxidase.

### Nematode proteins resembling yeast Fox2p

In *S. cerevisiae*, the second and third steps of classical *β*-oxidation are catalysed by Fox2p (Hiltunen *et al.*, 1992). From the outset, a computer search for Fox2p-like *C. elegans* proteins based on

Copyright © 2000 John Wiley & Sons, Ltd. 

Table 4. *C. elegans* proteins with similarities to *S. cerevisiae* classical $\beta$-oxidation enzymes

| | | | | | Subcellular location[b] | | | |
|---|---|---|---|---|---|---|---|---|
| Yeast | yC[a] | Worm | Similarity | wC[a] | pex | mit | cyt | Similar to[c] |
| Pox1p | INK | C48B4.1 | 1.4E-54 | SKL | 78 | 11 | 11 | Pox1p |
| | | F58F9.7 | 1.3E-52 | SKL | 78 | 11 | 11 | Pox1p |
| | | F08A8.2 | 1.0E-49 | AKL | 17 | 35 | 21 | Pox1p |
| | | F59F4.1 | 1.3E-41 | SKL | 78 | 11 | 11 | Pox1p |
| | | F25C8.1 | 1.0E-36 | AKM | 4 | 9 | 52 | Pox1p |
| | | F08A8.3 | 8.2E-36 | AKL | – | 43 | 26 | Pox1p |
| | | F08A8.1 | 1.6E-34 | SKL | 78 | 11 | 11 | Pox1p |
| | | F08A8.4 | 9.1E-34 | AKL | 44 | 22 | 22 | Pox1p |
| Fox2p | SKL | M03A8.1 | 5.9e-71 | SKL | 67 | 11 | 22 | Fox2p |
| | | E04F6.3 | 3.9e-44 | SKL | 78 | 11 | 11 | Fox2p |
| | | F09E10.3 | 3.2e-24 | FSM | – | 22 | 30 | Fox2p |
| | | Y39A1A.11 | 3.2e-18 | LGM | 9 | 4 | 65 | Fox2p |
| | | F54F3.4 | 6.4e-14 | RFS | – | 26 | 61 | Sps19p,Fox2p |
| | | D1054.8 | 5.8e-11 | LLH | – | 35 | 43 | YMR226c,Sps19p |
| | | T05F1.10 | 1.6e-10 | FHK | 4 | 22 | 22 | YMR226c |
| | | T02E1.5 | 1.9e-10 | RQA | 4 | 22 | 22 | YDL114w |
| | | C45B11.3 | 2.8e-10 | SKL | 78 | 11 | 11 | Fox2p |
| | | R05D8.b | 3.8e-10 | IDG | – | 22 | – | Sps19p |
| | | F01G4.2 | 4.4e-10 | MPA | 4 | 22 | 40 | YDL114w |
| | | T11F9.11 | 7.4e-10 | KDN | – | 11 | 11 | YDL114w,YMR226c |
| | | R05D8.e | 5.7e-09 | SSV | – | 30 | 26 | YMR226c,Sps19p |
| | | ZK829.1 | 1.3e-08 | AKA | 4 | 26 | 40 | YMR226c,YIR035c |
| | | F02C12.2 | 2.8e-07 | GKK | 4 | 40 | 26 | YMR226c |
| | | C17G10.8 | 3.9e-07 | GKL | 4 | 22 | 39 | Fox2p |
| Pot1p | IKE | F53A2.7 | 4.1E-54 | LGL | – | 26 | 52 | Erg10p,Pot1p |
| | | T02G5.8 | 3.2E-47 | QKL | – | 74 | 22 | Erg10p,Pot1p |
| | | T02G5.7 | 4.2E-45 | KKL | 9 | 35 | 40 | Erg10p,Pot1p |
| | | B0303.3 | 9.4E-39 | YGK | 9 | 56 | 9 | Pot1p,Erg10p |
| | | T02G5.4 | 1.3E-22 | QKL | 4 | 52 | 22 | Erg10p,Pot1p |
| | | Y57A10C.6 | 0.048 | SKI | 9 | 13 | 52 | Erg10p,Pot1p |

[a]yC refers to C termini of yeast proteins, wC to those in worm orthologues.
[b]Numbers reflect the percentage probability predicted by the PSORT II algorithm for a particular protein to be localized either in peroxisomes (pex), mitochondria (mit) or the cytoplasm (cyt). Columns for the localization in other subcellular sites, such as the nucleus, endoplasmic reticulum, plasma membrane, Golgi, etc., are not shown.
[c]refers to yeast proteins most similar to the worm orthologue.
– no score available.

complete peptide sequences was likely to reveal a large subset of proteins belonging to the extended family of non-metallo short-chain alcohol dehydrogenases. In this and subsequent sections, only those proteins with singular characteristics were addressed experimentally. The protein most similar to Fox2p (M03A8.1; Table 4) is denoted by WormPD™ as FAT-2 to indicate that it is probably a fatty acid desaturase. However, since it ends with SKL (Table 4) and is also highly similar to the mammalian trifunctional enzyme (WormPD™), it represents a good candidate for a peroxisomal $\beta$-oxidation protein. E04F6.3 (and to a lesser extent C45B11.3) similarly appears to fulfil the latter two criteria (Table 4).

The protein under the Fox2p section with the least similar value (C17G10.8; Table 4) is the product of the *fat-3* gene. Proteins with a Fox2p similarity below that of FAT-3 were not listed. FAT-3 is an interesting protein because it terminates with a GKL tripeptide that interacts with human and yeast Pex5p (Lametschwandtner *et al.*, 1998), and potentially also with *C. elegans* PEX-5 (uppermost tripeptide, third column, Table 3). To determine whether FAT-3 could represent a peroxisomal protein, a Gal4p–GKL from the screen by

Copyright © 2000 John Wiley & Sons, Ltd. 

Table 5. Two-hybrid interactions between *C. elegans* PEX-5 and PTS1-extended protein partners

| Strain | C terminus | *β*-Galactosidase activity[a] | Relative level |
|---|---|---|---|
| yAG1257 | M-HML[b] | 216.4±9.6 | 1.0 |
| yAG1256 | R-SKL | 574.5±23.2 | 2.7 |
| yAG1262 | W-AKL | 631.4±9.5 | 2.9 |
| yAG1263 | L-AKL | 446.8±22.2 | 2.1 |
| yAG1264 | F-AKL | 352.0±21.7 | 1.6 |
| yAG1265 | R-AKL | 1430.3±53.9 | 6.6 |
| yAG1257 | M-HML | 177.0±1.3 | 1.0 |
| yAG1256 | R-SKL | 934.9±89.0 | 5.3 |
| yAG1266 | A-AKM | 293.4±8.5 | 1.7 |
| yAG1257 | M-HML | 177.0±1.3 | 1.0 |
| yAG1256 | R-SKL | 934.9±89.0 | 5.3 |
| yAG1267 | Y-GKL | 90.3±2.4 | 0.5 |
| yAG1269 | R-GKL | 143.0±6.7 | 0.8 |
| yAG1268 | R-QKL | 57.9±4.6 | 0.3 |
| yAG1257 | M-HML | 221.6±5.5 | 1.0 |
| yAG1256 | R-SKL | 774.6±82.5 | 3.5 |
| yAG1270 | K-LKL | 3.8±0.3 | 0.0 |
| yAG1271 | D-LKL | 4.4±0.2 | 0.0 |

[a]nmol *O*-nitrophenyl-*β*-D-galactoside hydrolysed/min/mg protein; mean±SD, $n=3$.

[b]The amino acid to the left of the hyphen is adjacent to the C-terminal tripeptide and may be important for PTS1 function (Lametschwandtner *et al.*, 1998).

Lametschwandtner *et al.* (1998) was analysed for two-hybrid interaction with nematode PEX-5 (third cluster, Table 5). The results demonstrated that GKL gave rise to levels of *β*-galactosidase activities that were 0.5–0.8-fold of those obtained with HML (third cluster, Table 5). Therefore, the two-hybrid data for FAT-3 being a peroxisomal protein remain equivocal. FAT-2 (M03A8.1) and E04F6.3, on the other hand, might be promising as candidates for peroxisomal multifunctional enzymes.

### Putative nematode thiolases

The last step of the *β*-oxidation spiral in *S. cerevisiae* is catalysed by Pot1p/Fox3p (Einerhand *et al.*, 1991; Igual *et al.*, 1991), whose import depends on Pex7p (Marzioch *et al.*, 1994). *C. elegans* has been previously reported to contain Y57A10C.6/P-44 (Bun-Ya *et al.*, 1997), a peroxisomal type-II 3-oxoacyl-CoA thiolase (Table 4). However, P-44 is similar to mammalian sterol carrier protein x (SCPx) and, therefore, does not represent a classical *β*-oxidation thiolase. Instead, it is thought to be involved in phytanic acid degradation and bile acid synthesis (Bun-Ya *et al.*, 1997; Maebuchi *et al.*, 1999).

The C termini of the Pot1p/Fox3p-like potential *β*-oxidation thiolases identified here (Table 4) did not look like PTS1s, with the exception of QKL (extending from T02G5.4 and T02G5.8), which has previously been shown to interact with yeast Pex5p (Lametschwandtner *et al.*, 1998) and possibly KKL (extending from T02G5.7). Our two-hybrid results showed that QKL was even less efficient than GKL as a nematode PEX-5 interaction partner, since it gave rise to *β*-galactosidase values that were only 0.3-fold of those generated using HML (third cluster, Table 5). An appropriate Gal4p fusion to determine whether KKL might be efficient at interacting with nematode PEX-5 was not available. This is because Gal4p–KKL had not turned up as a positive interaction partner, either in the present two-hybrid screen (Table 3) or in previous ones (Kragler *et al.*, 1998; Lametschwandtner *et al.*, 1998). The possibility of KKL interacting with PEX-5 notwithstanding, the apparent absence of a potential nematode thiolase with an efficient PTS1 could mean that, like the situation in *S. cerevisiae* (and mammals), the putative *C. elegans* thiolase is also imported in a PEX-5-independent manner.

### Auxiliary enzymes of *β*-oxidation: potential nematode 2,4-reductases

From the data gathered on nematode Pox1p- and Fox2p orthologues, it is possible that *C. elegans* contains the components of a basic peroxisomal *β*-oxidation spiral for degrading saturated fatty acids. To break down unsaturated fatty acids with odd-numbered double bonds such as oleic acid (*cis*-$C_{18:1(9)}$), yeast engage peroxisomal Eci1p (Gurvitz *et al.*, 1998; Geisbrecht *et al.*, 1998), corresponding to the auxiliary enzyme $\Delta^3$-$\Delta^2$-enoyl-CoA isomerase. Eci1p is also critical for breaking down even-numbered *cis*-double bonds in fatty acids such as petroselinic acid (*cis*-$C_{18:1(6)}$). Metabolism of petroselinic acid additionally requires Sps19p (Gurvitz *et al.*, 1997) that represents the auxiliary enzyme 2,4-dienoyl-CoA reductase (2,4-reductase). In mammals, 2,4-reductases are thought to also participate in degrading oleic acid (Smeland *et al.*, 1992).

A computer search for Sps19p orthologues revealed three well-scoring proteins, two of which (T05C12.3 and W01C9.4) were predicted to be

Copyright © 2000 John Wiley & Sons, Ltd. 

mitochondrial, and the one terminating with SKL (F53C11.3) to be peroxisomal (Table 6). Like the previous situation with Fox2p, a search for Sps19p-like nematode proteins was bound to reveal additional less-related dehydrogenases. For example, several of the worm proteins identified here are similar to other putative yeast dehydrogenases (Ymr226cp, Yir035cp, and Ydl114wp; right column, Table 6) which, however, do not represent 2,4-reductases, since the yeast *sps19*Δ mutant does not contain redundant isoenzymes allowing it to utilize petroselinic acid (Gurvitz *et al.*, 1997). The SKL tripeptide extending from F53C11.3 has been amply demonstrated in the present work to interact with *C. elegans* PEX-5 (Tables 3, 5). On the other hand, the tripeptides EKP and EKA extending from T05C12.3 and W01C9.4 (Table 6) do not conform to the PTS1 consensus and have not been isolated as Pex5p-interacting tripeptides in two-hybrid screens (Lametschwandtner *et al.*, 1998). The lack of a canonical PTS1 in these two proteins was coincidental with a high probability for a mitochondrial location. The important implication of the putative distribution of the conjectured nematode 2,4-reductases to both peroxisomes and mitochondria is discussed in a later section.

### Nematode hydratase/isomerase proteins

Eci1p is 46% identical to Dci1p (Geisbrecht *et al.*, 1999a) which represents peroxisomal $\Delta^{3,5}$-$\Delta^{2,4}$-dienoyl-CoA isomerase (Gurvitz *et al.*, 1999a). Dci1p contains a PTS1-like HKL C terminus but, like the HRL-terminating Eci1p, could be demonstrated to be imported into peroxisomes by an internal signal acting via a novel route (Karpichev and Small, 2000). Of the five potential nematode Eci1p or Dci1p orthologues shown (Table 6), only one

Table 6. *C. elegans* proteins with similarities to *S. cerevisiae* β-oxidation auxiliary enzymes

| | | | | | Subcellular location | | | |
|---|---|---|---|---|---|---|---|---|
| Yeast | yC | Worm | Similarity | wC | pex | mit | cyt | Similar to |
| Sps19p | SKL | T05C12.3 | 1.2e-29 | EKP | 4 | 48 | 30 | Sps19p |
| | | W01C9.4 | 8.8e-29 | EKA | – | 65 | 13 | Sps19p |
| | | F53C11.3 | 3.8e-28 | SKL | 78 | 11 | 11 | Sps19p |
| | | R05D8.b | 8.7e-22 | IDG | – | 22 | – | Sps19p |
| | | R05D8.d | 1.8e-21 | TAR | 4 | 9 | 13 | Sps19p |
| | | D1054.8 | 1.6e-20 | LLH | – | 35 | 43 | YMR226c,Sps19p |
| | | T01G6.1 | 1.6e-20 | LTG | – | 56 | 35 | YMR226c,Sps19p |
| | | F28H7.2 | 4.9e-19 | KEL | – | 39 | 43 | YMR226c |
| | | R05D8.c | 6.3e-19 | SQE | – | 35 | – | YMR226c,Sps19p |
| | | C01G12.5 | 1.0e-18 | LKH | – | 65 | 22 | YMR226c,Sps19p |
| | | F25D1.5 | 1.3e-18 | LSQ | – | 35 | 48 | YMR226c,Sps19p |
| | | F54F3.4 | 5.7e-18 | RFS | – | 26 | 61 | Sps19p,Fox2p |
| | | C06E4.3 | 7.2e-18 | PQK | 4 | – | 35 | YMR226c,Sps19p |
| | | F26D2.15 | 1.2e-16 | LKA | 4 | 35 | 35 | YMR226c,Sps19p |
| | | R05D8.e | 2.3e-16 | SSV | – | 30 | 26 | YMR226c,Sps19p |
| | | F09E10.3 | 7.2e-16 | FSM | – | 26 | 48 | Fox2p |
| | | R08H2.1 | 1.4e-15 | LNQ | 4 | 35 | 35 | YMR226c |
| | | ZK829.1 | 4.1e-14 | AKA | 4 | 26 | 48 | YMR226c,YIR035c |
| | | Y39A1A.11 | 5.6e-14 | LGM | 4 | – | 61 | Fox2p |
| | | F02C12.2 | 2.2e-13 | GKK | 4 | 43 | 26 | YMR226c |
| | | C06E4.6 | 2.7e-13 | PRQ | 4 | 17 | 43 | YMR226c |
| | | W03F9.9 | 1.4e-12 | RAE | – | 9 | 65 | YMR226c |
| | | F01G4.2 | 6.5e-12 | MPA | 4 | 4 | 52 | YDL114w |
| | | M03A8.1 | 3.3e-11 | SKL | 56 | 22 | 22 | Fox2p |
| Eci1p | HRL | R06F6.9 | 1.6E-20 | AKK | – | 44 | 26 | E/Dci1p,Acb1p |
| Dci1p | HKL | T05G5.6 | 9.3E-10 | ESK | – | 52 | 30 | YDR036c,Eci1p |
| | | F58A6.1 | 4.5E-07 | SKL | 78 | 11 | 11 | YDR036c,Eci1p |
| | | B0272.4 | 7.9E-07 | SKI | – | 11 | 11 | YDR036c,E/Dci1p |
| | | F38H4.8 | 7.4E-05 | KKN | – | 91 | 4 | YDR036c,E/Dci1p |

Copyright © 2000 John Wiley & Sons, Ltd.

contained an obvious PTS1 (F58A6.1), whereas the remaining proteins were predicted as being predominantly mitochondrial. Although SKI acts as PTS1 for *Hansenula polymorpha* catalase (Didion and Roggenkamp, 1992), SKI and AKK are not considered as potential PTS1s in *S. cerevisiae* or humans (Lametschwandtner *et al.*, 1998).

Dci1p and Eci1p belong to the low similarity hydratase/isomerase family of proteins, all of which act on CoA-ester substrates and some are additionally entrained in *β*-oxidation (Müller-Newen *et al.*, 1995). Other members of this family include rat mitochondrial/peroxisomal ECH1 (Filppula *et al.*, 1998) and peroxisomal MFE type 1 (Palosaari and Hiltunen, 1990), naphthoate synthase, 4-chlorobenzoyl-CoA dehalogenase, carnitine racemase, 3-hydroxyisobutyryl-CoA hydrolase (Müller-Newen *et al.*, 1995), and *S. cerevisiae* Ydr036cp (Gurvitz *et al.*, 1999a). An additional computer search for further hydratase/isomerase proteins based on similarity to Ydr036cp (Table 7) revealed at least two nematode proteins that might represent peroxisomal $\Delta^3$-$\Delta^2$-enoyl-CoA isomerases: F09F7.4 ending with LKL, and the SKL-terminating protein F43H9.1. However, these data are not sufficient to substantiate our theory that nematodes might contain the requisite process for degrading oleic acid.

*C. elegans* F09F7.4 and *S. cerevisiae* Ydr036cp, found when searching for Eci1p and Dci1p orthologues, are the two most similar proteins in the respective species (45 and 33%, respectively) to human 3-hydroxyisobutyryl-CoA hydrolase (Hawes *et al.*, 1996). A deficiency in this enzyme is lethal to humans (Brown *et al.*, 1982), and a decreased level of this activity is thought to be a cause of liver failure in cirrhosis (Taniguchi *et al.*, 1996). Like the human enzyme, which is located in the mitochondria, the putative nematode protein was predicted by the algorithm to be similarly mitochondrial. However, this novel protein additionally terminates with LKL which, at least in *S. cerevisiae,* represents a Pex5p-interacting PTS1 (Lametschwandtner *et al.*, 1998). To determine whether LKL could represent a functional PTS1 in *C. elegans*, it was assayed for two-hybrid interaction (Table 5). The results showed that LKL was significantly less efficient compared to SKL or even HML in interacting with nematode PEX-5, yielding *β*-galactosidase activities that were essentially below the detection level of the system used (bottom panel, Table 5). We therefore reaffirm the algorithm's prediction that F09F7.4 is an extra-peroxisomal protein.

Table 7. *YDR036c*-like *C. elegans* proteins with a hydratase/isomerase fingerprint

| Worm | Similarity | wC | Subcellular location: pex | mit | cyt | Similar to |
|---|---|---|---|---|---|---|
| F09F7.4 | 7.5e-39 | LKL | 4 | 48 | 35 | YDR036c |
| F56B3.5 | 1.8e-14 | KRD | 9 | 44 | 39 | YDR036c |
| C29F3.1 | 9.5e-11 | FYS | – | 100 | – | YDR036c |
| T08B2.7 | 5.4e-10 | FYN | – | 22 | 48 | YDR036c |
| F43H9.1 | 5.1e-09 | SKL | 67 | 11 | 22 | YDR036c |
| Y25C1A.a | 4.9e-06 | EDV | – | 65 | 17 | YDR036c, E/Dci1p |

### Discussion

Combining experimental data with results from computer searches offers a powerful method for making meaningful predictions regarding the function of novel proteins. Here, we wanted to know whether *C. elegans* contains the full set of peroxisomal *β*-oxidation enzymes analogous to that found in *S. cerevisiae*, including those enzymes essential for degrading unsaturated fatty acids. For this purpose, we have identified nematode PEX-5. This meant that *C. elegans* contains at least one component of the peroxisomal import machinery known to exist in other organisms, including yeast, plants and mammals. Such homology studies using yeast *PEX* genes have been instrumental in identifying the corresponding human genes (Subramani, 1997). By following a similar approach, homology probing should allow the isolation of all the potential *C. elegans* peroxin genes (i.e. involved in peroxisome biogenesis). We have also identified potential orthologues of yeast proteins involved in *β*-oxidation. Since several of these orthologues terminated with a PTS1 that could interact with nematode PEX-5 in the yeast two-hybrid assay, we predict that at least some of them represent peroxisomal *β*-oxidation proteins.

Regarding those orthologues not containing an efficient PEX-5-interacting C terminus, such as LKL or GKL (F09F7.4 and FAT-3, respectively), it should be noted that in certain yeast peroxisomal proteins PTS1 occurs in a redundant system with additional cryptic PTSs. Examples for this are given

Copyright © 2000 John Wiley & Sons, Ltd. 

by Cta1p (Kragler *et al.*, 1993), Cat2p (Elgersma *et al.*, 1995), Eci1p (Karpichev and Small, 2000) and Dci1p (Karpichev and Small, 2000). Therefore, although we do not provide positive evidence for this, we hypothesize that in such cases of type 1 signals interacting only weakly with nematode PEX-5, these could be augmented by internal signals. It is also not clear whether nematodes actually have PTS2-containing proteins or the cognate receptor, PEX-7. A clue to the existence of PTS2 proteins could have come from the identification of PTS1-less proteins with a high probability for peroxisomal localization. However, inspection of Tables 4 and 6 does not reveal any PTS2 proteins (including the putative thiolases), since all those orthologues to which were attributed high probabilities for a peroxisomal location were actually terminated with a canonical PTS1. In addition, a search of WormPD$^{TM}$ for a potential PEX-7 revealed a very large protein, Y32H12A.8 (and its putative paralogue Y32H12A_57.A), consisting of 3885 amino acids with only a weak similarity (26% identity over 246 amino acids) to the 323 amino acid-long human PEX7. Therefore, the issue of the existence of a PEX-7-dependent peroxisomal import mechanism remains open for the time being.

As proposed in the Introduction, were (the putative peroxisomal) $\beta$-oxidation in *C. elegans* to be augmented by a mitochondrial process, such as occurs in mammals but not yeast or plants (Kunau *et al.*, 1995), nematodes could prove to be a better model than *S. cerevisiae* for studying the relationship between the two compartmentalized processes. A principal difference between the two processes is that the first step in peroxisomal $\beta$-oxidation is initiated by an acyl-CoA oxidase, whereas that in mitochondria is executed by an acyl-CoA dehydrogenase. A search for proteins with similarities to acyl-CoA dehydrogenase revealed 13 potential mitochondrial proteins (WormPD$^{TM}$). The PSORT II results presented here, predicting that at least some of the yeast-like $\beta$-oxidation orthologues contained mitochondrial leader signals, is also a fairly good indication that nematodes might resemble mammals in having an additional mitochondrial $\beta$-oxidation compartment. This could extend the nematode's potential versatility for studying human disorders associated with deficient mitochondrial $\beta$-oxidation.

For example, human 2,4-dienoyl-CoA reductase deficiency (OMIM number 222745; 24 February 2000) is thought to be due to a defective mitochondrial 2,4-reductase (Roe *et al.*, 1990). A candidate human mitochondrial enzyme has been identified (Koivuranta *et al.*, 1994) and shown to be a physiological 2,4-reductase devoid of any cryptic PTSs (Gurvitz *et al.*, 1999b). Although a yeast 2,4-reductase knockout has been generated (Gurvitz *et al.*, 1997), this mutant fails to serve as an appropriate model for studying the underlying mechanism behind this deficiency, for two reasons. First, the missing yeast enzyme is peroxisomal, and second, unlike the human patient, which could not degrade odd-numbered *cis*-double bonds properly (Roe *et al.*, 1990), the mutant yeast strain is not affected for this function (Gurvitz *et al.*, 1997).

The distribution of the putative 2,4-reductases in the nematode is reminiscent of that in mammals (Table 6); rodents and probably also humans contain one peroxisomal 2,4-reductase (Fransen *et al.*, 1999; Geisbrecht *et al.*, 1999b) and two mitochondrial isoenzymes (Hakkola *et al.*, 1989; Hakkola and Hiltunen, 1993). Hence, in light of the limitations of using yeast cells which have only peroxisomal $\beta$-oxidation, *C. elegans* might prove to be a more appropriate test organism for examining the effect of knocking out the mitochondrial 2,4-reductases on growth on oleic acid. Although several mouse knockouts for $\beta$-oxidation enzymes exist, the effect of removing the respective proteins in worms might be more easily studied.

### Acknowledgements

Our gratitude goes to Leila Wabnegger for technical help. We are especially grateful to Dr Hanspeter Rottensteiner and Professor Kalervo Hiltunen for critically reading the manuscript. This work was supported by the Fonds zur Förderung der wissenschaftlichen Forschung (FWF), Vienna, Austria (Grants P12061-MOB to H.R. and B.H. and P12118-MOB to A.H.).

### References

Altschul SF, Madden TL, Schaffer AA, *et al.* 1997. Gapped BLAST and PSI-BLAST: a new generation of protein database search programs. *Nucleic Acids Res* **25**: 3389–3402.

Bartel P, Chien CT, Sternglanz R, Fields S. 1993. Elimination of false positives that arise in using the two-hybrid system. *Biotechniques* **14**: 920–924.

Bradford MM. 1976. A rapid and sensitive method for the

Copyright © 2000 John Wiley & Sons, Ltd. 

quantitation of microgram quantities of protein utilizing the principle of protein-dye binding. *Anal Biochem* **72**: 248–254.

Brown GK, Hunt SM, Scholem R, *et al.* 1982. β-Hydroxyisobutyryl-coenzyme A deacylase deficiency: a defect in valine metabolism associated with physical malformations. *Pediatrics* **70**: 532–538.

Bun-Ya M, Maebuchi M, Hashimoto T, Yokota S, Kamiryo T. 1997. A second isoform of 3-ketoacyl-CoA thiolase found in *Caenorhabditis elegans*, which is similar to sterol carrier protein x but lacks the sequence of sterol carrier protein 2. *Eur J Biochem* **245**: 252–259.

Chen D-C, Yang B-C, Kuo T-T. 1992. One-step transformation of yeast in stationary phase. *Curr Genet* **21**: 83–84.

Chervitz SA, Aravind L, Sherlock G, *et al.* 1998. Comparison of the complete protein sets of worm and yeast: orthology and divergence. *Science* **282**: 2022–2028.

Chevray PM, Nathans D. 1992. Protein interaction cloning in yeast: identification of mammalian proteins that react with the leucine zipper of Jun. *Proc Natl Acad Sci U S A* **89**: 5789–5793.

Consortium. 1998. Genome sequence of the nematode *C. elegans*: a platform for investigating biology. *Science* **282**: 2012–2018.

Costanzo MC, Hogan JD, Cusick ME, *et al.* 2000. The yeast proteome database. YPD and *Caenorhabditis elegans* proteome database. WormPD: comprehensive resources for the organization and comparison of model organism protein information. *Nucleic Acids Res* **28**: 73–76.

de Duve C, Baudhuin P. 1966. Peroxisomes. Microbodies and related particles. *Physiol Rev* **46**: 323–357.

Didion T, Roggenkamp R. 1992. Targeting signal of the peroxisomal catalase in the methylotrophic yeast *Hansenula polymorpha*. *FEBS Lett* **303**: 113–116.

Dmochowska A, Dignard D, Maleszka R, Thomas DY. 1990. Structure and transcriptional control of the *Saccharomyces cerevisiae POX1* gene encoding acyl-coenzyme A oxidase. *Gene* **88**: 247–252.

Einerhand AW, Voorn-Brouwer TM, Erdmann R, Kunau W-H, Tabak HF. 1991. Regulation of transcription of the gene coding for peroxisomal 3-oxoacyl-CoA thiolase of *Saccharomyces cerevisiae*. *Eur J Biochem* **200**: 113–122.

Elgersma Y, van Roermund CW, Wanders RJ, Tabak HF. 1995. Peroxisomal and mitochondrial carnitine acetyltransferases of *Saccharomyces cerevisiae* are encoded by a single gene. *EMBO J* **14**: 3472–3479.

Elgersma Y, Vos A, van den Berg M, van Roermund CW, *et al.* 1996. Analysis of the carboxy-terminal peroxisomal targeting signal 1 in a homologous context in *Saccharomyces cerevisiae*. *J Biol Chem* **271**: 26375–26382.

Filppula SA, Yagi AI, Kilpeläinen SH, *et al.* 1998. $\Delta^{3,5}$-$\Delta^{2,4}$-Dienoyl-CoA isomerase from rat liver. Molecular characterization. *J Biol Chem* **273**: 349–355.

Fransen M, Van Veldhoven PP, Subramani S. 1999. Identification of peroxisomal proteins by using M13 phage protein VI phage display: molecular evidence that mammalian peroxisomes contain a 2,4-dienoyl-CoA reductase. *Biochem J* **340**: 561–568.

Geisbrecht BV, Zhu D, Schulz K, *et al.* 1998. Molecular characterization of *Saccharomyces cerevisiae* $\Delta^3$,$\Delta^2$-enoyl-CoA isomerase. *J Biol Chem* **273**: 33184–33191.

Geisbrecht BV, Schulz K, Nau K, *et al.* 1999a. Preliminary characterization of Yor180Cp: identification of a novel peroxisomal protein of *Saccharomyces cerevisiae* involved in fatty acid metabolism. *Biochem Biophys Res Commun* **260**: 28–34.

Geisbrecht BV, Liang X, Morrell JC, Schulz H, Gould SJ. 1999b. The mouse gene *PDCR* encodes a peroxisomal $\Delta^2$,$\Delta^4$-dienoyl-CoA reductase. *J Biol Chem* **274**: 25814–25820.

Gould SJ, Keller GA, Subramani S. 1987. Identification of a peroxisomal targeting signal at the carboxy-terminus of firefly luciferase. *J Cell Biol* **105**: 2923–2931.

Gould SJ, Keller GA, Schneider M, *et al.* 1990. Peroxisomal protein import is conserved between yeast, plants, insects and mammals. *EMBO J* **9**: 85–90.

Gurvitz A, Rottensteiner H, Kilpeläinen SH, *et al.* 1997. The *Saccharomyces cerevisiae* peroxisomal 2,4-dienoyl-CoA reductase is encoded by the oleate-inducible gene *SPS19*. *J Biol Chem* **272**: 22140–22147.

Gurvitz A, Mursula AM, Firzinger A, *et al.* 1998. Peroxisomal $\Delta^3$-*cis*-$\Delta^2$-*trans*-enoyl-CoA isomerase encoded by *ECI1* is required for growth of the yeast *Saccharomyces cerevisiae* on unsaturated fatty acids. *J Biol Chem* **273**: 31366–31374.

Gurvitz A, Mursula AM, Yagi AI, *et al.* 1999a. Alternatives to the isomerase-dependent pathway for the β-oxidation of oleic acid are dispensable in *Saccharomyces cerevisiae*. Identification of *YOR180c/DCI1* encoding peroxisomal $\Delta^{3,5}$-$\Delta^{2,4}$-dienoyl-CoA isomerase. *J Biol Chem* **274**: 24514–24521.

Gurvitz A, Wabnegger L, Yagi AI, *et al.* 1999b. Function of human mitochondrial 2,4-dienoyl-CoA reductase and rat monofunctional $\Delta^3$-$\Delta^2$-enoyl-CoA isomerase during peroxisomal β-oxidation in *Saccharomyces cerevisiae*. *Biochem J* **344**: 903–914.

Hakkola EH, Autio-Harmainen HI, Sormunen RT, Hassinen IE, Hiltunen JK. 1989. The known purified mammalian 2,4-dienoyl-CoA reductases are mitochondrial isoenzymes. *J Histochem Cytochem* **37**: 1863–1867.

Hakkola EH, Hiltunen JK. 1993. The existence of two mitochondrial isoforms of 2,4-dienoyl-CoA reductase in the rat. *Eur J Biochem* **215**: 199–204.

Hawes JW, Jaskiewicz J, Shimomura Y, *et al.* 1996. Primary structure and tissue-specific expression of human β-hydroxyisobutyryl-coenzyme A hydrolase. *J Biol Chem* **271**: 26430–26434.

Hiltunen JK, Wenzel B, Beyer A, Erdmann R, Fosså A, Kunau W-H. 1992. Peroxisomal multifunctional β-oxidation protein of *Saccharomyces cerevisiae.* Molecular analysis of the *FOX2* gene and gene product. *J Biol Chem* **267**: 6646–6653.

Igual JC, Matallana E, Gonzalez-Bosch C, Franco L, Perez-Ortin JE. 1991. A new glucose-repressible gene identified from the analysis of chromatin structure in deletion mutants of yeast *SUC2* locus. *Yeast* **7**: 379–389.

Itoh M, Suzuki Y, Akaboshi S, Zhang Z, Miyabara S, Takashima S. 2000. Developmental and pathological expression of peroxisomal enzymes: their relationship of D-bifunctional protein deficiency and Zellweger syndrome. *Brain Res* **858**: 40–47.

Jardim A, Liu W, Zheleznova E, Ullman B. 2000. Peroxisomal targeting signal-1 receptor protein PEX5 from *Leishmania donovani*. Molecular, biochemical, and immunocytochemical characterization. *J Biol Chem* **275**: 13637–13644.

Karpichev IV, Small GM. 2000. Evidence for a novel pathway for the targeting of a *Saccharomyces cerevisiae* peroxisomal

Copyright © 2000 John Wiley & Sons, Ltd. 

protein belonging to the isomerase/hydratase family. *J Cell Sci* **113**: 533–544.

Koivuranta KT, Hakkola EH, Hiltunen JK. 1994. Isolation and characterization of cDNA for human 120 kDa mitochondrial 2,4-dienoyl-coenzyme A reductase. *Biochem J* **304**: 787–792.

Kragler F, Lametschwandtner G, Christmann J, Hartig A, Harada JJ. 1998. Identification and analysis of the plant peroxisomal targeting signal 1 receptor NtPEX5. *Proc Natl Acad Sci U S A* **95**: 13336–13341.

Kragler F, Langeder A, Raupachova J, Binder M, Hartig A. 1993. Two independent peroxisomal targeting signals in catalase A of *Saccharomyces cerevisiae*. *J Cell Biol* **120**: 665–673.

Kunau W-H, Dommes V, Schulz H. 1995. $\beta$-Oxidation of fatty acids in mitochondria, peroxisomes, and bacteria: a century of continued progress. *Prog Lipid Res* **34**: 267–342.

Lametschwandtner G, Brocard C, Fransen M, Van Veldhoven P, Berger J, Hartig A. 1998. The difference in recognition of terminal tripeptides as peroxisomal targeting signal 1 between yeast and human is due to different affinities of their receptor Pex5p to the cognate signal and to residues adjacent to it. *J Biol Chem* **273**: 33635–33643.

Maebuchi M, Togo SH, Yokota S, Ghenea S, Bun-Ya M, Kamiryo T, Kawahara A. 1999. Type-II 3-oxoacyl-CoA thiolase of the nematode *Caenorhabditis elegans* is located in peroxisomes, highly expressed during larval stages and induced by clofibrate. *Eur J Biochem* **264**: 509–515.

Marzioch M, Erdmann R, Veenhuis M, Kunau W-H. 1994. *PAS7* encodes a novel yeast member of the WD-40 protein family essential for import of 3-oxoacyl-CoA thiolase, a PTS2-containing protein, into peroxisomes. *EMBO J* **13**: 4908–4918.

McNew JA, Goodman JM. 1996. The targeting and assembly of peroxisomal proteins: some old rules do not apply. *Trends Biochem Sci* **21**: 54–58.

Miller JH. 1972. *Experiments in Molecular Genetics*, Cold Spring Harbor Laboratory Press: New York.

Müller-Newen G, Janssen U, Stoffel W. 1995. Enoyl-CoA hydratase and isomerase form a superfamily with a common active-site glutamate residue. *Eur J Biochem* **228**: 68–73.

Palosaari PM, Hiltunen JK. 1990. Peroxisomal bifunctional protein from rat liver is a trifunctional enzyme possessing 2-enoyl-CoA hydratase, 3-hydroxyacyl-CoA dehydrogenase, and $\Delta^3,\Delta^2$-enoyl-CoA isomerase activities. *J Biol Chem* **265**: 2446–2449.

Rehling P, Marzioch M, Niesen F, Wittke E, Veenhuis M, Kunau W-H. 1996. The import receptor for the peroxisomal targeting signal 2. PTS2 in *Saccharomyces cerevisiae* is encoded by the *PAS7* gene. *EMBO J* **15**: 2901–2913.

Roe CR, Millington DS, Norwood DL, *et al*. 1990. 2,4-Dienoyl-coenzyme A reductase deficiency: a possible new disorder of fatty acid oxidation. *J Clin Invest* **85**: 1703–1707.

Sambrook J, Fritsch EF, Maniatis T. 1989. *Molecular Cloning: A Laboratory Manual*, 2nd edn. Cold Spring Harbor Laboratory Press: New York.

Smeland TE, Nada M, Cuebas D, Schulz H. 1992. NADPH-dependent $\beta$-oxidation of unsaturated fatty acids with double bonds extending from odd-numbered carbon atoms. *Proc Natl Acad Sci U S A* **89**: 6673–6677.

Sommer JM, Cheng QL, Keller G-A, Wang CC. 1992. *In vivo* import of firefly luciferase into the glycosomes of *Trypanosoma brucei* and mutational analysis of the C-terminal targeting signal. *Mol Biol Cell* **3**: 749–759.

Subramani S. 1993. Protein import into peroxisomes and biogenesis of the organelle. *Ann Rev Cell Biol* **9**: 445–478.

Subramani S. 1997. *PEX* genes on the rise. *Nature Genet* **15**: 331–333.

Sulston JE, Schierenberg E, White JG, Thomson JN. 1983. The embryonic cell lineage of the nematode *Caenorhabditis elegans*. *Dev Biol* **100**: 64–119.

Taniguchi K, Nonami T, Nakao A, *et al*. 1996. The valine catabolic pathway in human liver: effect of cirrhosis on enzyme activities. *Hepatology* **24**: 1395–1398.

Togo SH, Maebuchi M, Yokota S, Bun-Ya M, Kawahara A, Kamiryo T. 2000. Immunological detection of alkaline-diaminobenzidine-negative peroxisomes of the nematode *Caenorhabditis elegans*. Purification and unique pH optima of peroxisomal catalase. *Eur J Biochem* **267**: 1307–1312.

Van der Leij I, Franse MM, Elgersma Y, Distel B, Tabak HF. 1993. PAS10 is a tetratricopeptide-repeat protein that is essential for the import of most matrix proteins into peroxisomes of *Saccharomyces cerevisiae*. *Proc Natl Acad Sci U S A* **90**: 11782–11786.

Wang H, Le Dall MT, Wache Y, Laroche C, Belin JM, Nicaud JM. 1999. Cloning, sequencing, and characterization of five genes coding for acyl-CoA oxidase isozymes in the yeast *Yarrowia lipolytica*. *Cell Biochem Biophys* **31**: 165–174.

Yang M, Wu Z, Fields S. 1995. Protein–peptide interactions analyzed with the yeast two-hybrid system. *Nucleic Acids Res* **23**: 1152–1156.

Copyright © 2000 John Wiley & Sons, Ltd.
